# Prolonged Photosensitivity Following Exposure to Glyphosate Herbicide

**DOI:** 10.1111/phpp.70115

**Published:** 2026-07-28

**Authors:** Jean Ayer, Donna Parkin, Lesley E. Rhodes

**Affiliations:** ^1^ Photobiology Unit, Dermatology Centre, Salford Royal Hospital Northern Care Alliance NHS Foundation Trust, Manchester Academic Health Science Centre Manchester UK; ^2^ Division of Musculoskeletal and Dermatological Sciences, School of Biological Sciences, Faculty of Biology, Medicine and Health, NIHR Manchester Biomedical Research Centre University of Manchester Manchester UK

**Keywords:** persistent light reaction, photosensitisation, photosensitivity, ultraviolet radiation, weedkiller

## 1 Introduction

Prolonged or persistent light reaction (PLR) after exposure to an exogenous agent is a rarely reported phenomenon [1, 2]. We present a novel case of severe prolonged photosensitivity to ultraviolet (UV) radiation, with onset in a father and daughter showing a clear temporal relationship to glyphosate‐containing herbicide (weedkiller) exposure.

## 2 Case Report

A 33‐year‐old man and his 4‐year‐old daughter, both white Caucasian, were referred for photoinvestigation with simultaneous onset of a photodistributed dermatitis. A history was given of exposure to herbicide on a sunny, breezy day in August 2022, whilst walking near council‐owned land. A council worker on a quad bike and wearing covering protective clothing was seen spraying herbicide when father and child were ~5 m downwind. They felt droplets of the spray on their exposed skin sites.

**FIGURE 1:**
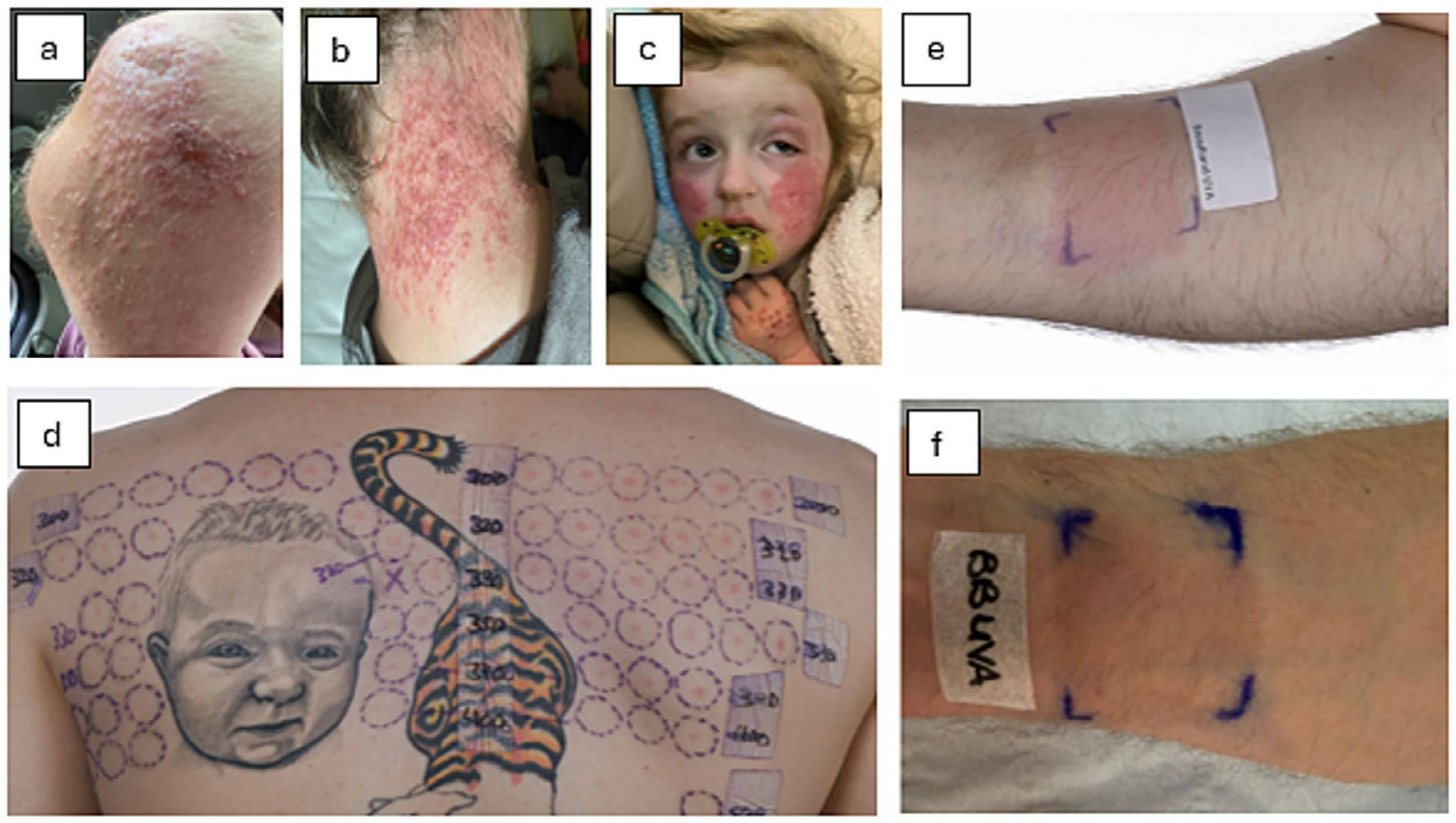
Photodistributed eruption—severe erythematous, scaly, papulovesicular eruption: (a) Father's dorsal arm (b) Father's posterior neck, (c) Daughter's face, and Phototesting features: (d) Father's initial monochromator phototesting (April 2023), showing low minimal erythema doses to UVB and UVA wavelengths across 300–370 nm; doses administered increase in a horizontal row from left to right with a diversion to avoid the tattoo, (e) Father's initial broadband UVA (BBUVA) provocation response (April 2023) – severe papular erythema, and (f) Father's latest BBUVA provocation response (November 2025) – mild papular erythema.

Overnight, both father and child experienced severe pruritus, and 3 days post‐exposure they had developed a dramatic erythematous, scaly, papulovesicular eruption confined to photo‐exposed areas, including face, neck, dorsal hands, elbows, forearms and lower legs to the edge of their clothing (Figure 1a–c). The initial eruption settled in 3–5 days, but following further sun exposures of 15–30 min, they experienced rapid and severe recurrences, with major continuing impact on their lives.

Detailed photoinvestigation was performed, modified due to the child's young age. This included monochromator phototesting on the back to narrow bandwidths of radiation, to determine their minimal erythemal doses (MEDs) at 24 h. Additionally, photo‐provocation testing was performed to limited areas of the forearms with solar simulated UV radiation (SSR) and broadband UVA (BBUVA). The first phototesting procedures, in 2023, showed severe photosensitivity in both patients, with reduced MEDs to UVB and UVA through 300–370 nm (Figure 1d, Table 1). After one exposure with either provocation source, the father developed a severe localised eruption (Figure 1e, Table 1), and the child showed papular erythema (Table 1).

**TABLE 1:** Monochromator and provocation phototesting results over time in Father and Daughter.

Father				Daughter		
Monochromator phototesting Wavelength (bandwidth) nm	Test 1 Apr 2023 MED (J/cm^2^)^a^	Test 2 Nov 2023 MED (J/cm^2^)^a^	Test 3 Feb 2025 MED (J/cm^2^)^a^	Monochromator phototesting Wavelength (bandwidth) nm	Test 1 June 2023 MED (J/cm^2^)^a^	Test 2 July 2024 MED (J/cm^2^)^a^
300 (10)	1.3↓	1.8↓	5↓	300 (10)	7↓	Neg > 14
320 (10)	0.13↓	0.13↓	0.25↓	320 (10)	0.7↓	Neg > 1
330 (10)	0.44↓	0.22↓	1.3↓			
350 (10)	3.5↓	1.8↓	5↓	350 (10)	5↓	Neg > 10
370 (15)	14↓	3.5↓	14↓	370 (15)	14↓	Neg > 20
400 (15)	57	40	57	400 (15)	Neg > 40	Neg > 40
500 (25)	Neg > 50	Neg > 50	Neg > 50			
600 (25)	Neg > 50	Neg > 50	Neg > 50			
**Provocation phototesting**						
BBUVA	Severe confluent erythema, papules & oedema	Severe confluent erythema, papules & oedema	Mild erythema & papules	BBUVA	Mild erythema & papules	? Faint erythema—borderline
SSR	Severe confluent erythema, papules & oedema	Severe confluent erythema, papules & oedema	Mild erythema	SSR	Mild erythema & papules	Neg

*Note:* Modified monochromator phototesting (fewer wavelengths and doses) was performed in the Daughter in view of her young age. Arrows indicate reduced minimal erythema doses (MEDs) compared with the normal range.

Abbreviations: BBUVA, Broadband UVA; SSR, Solar simulated UVR.

^a^ MEDs are in J/cm^2^ except at 300 nm where they are mJ/cm^2^.

Patch and photopatch testing was performed in both patients. This included patch testing to the standard, plant, fragrance, and face series, and photopatch with control patch testing to methylisothiazolinone (MIT, 0.2%), benzothiazolinone (BIT, 0.1%), and octyisothiazolinone (OIT, 0.1%) as these can be used as coformulants with glyphosate weedkiller. Patches were applied for 48 h, before BBUVA exposure (5 J/cm^2^ in father, 2.5 J/cm^2^ in child). Apart from a (+) positive photopatch test to benzalkonium chloride in the father, interpreted as incidental, there were no positive patch or photopatch tests. Detailed composition of the glyphosate formulation was not forthcoming from the council, and the father declined testing with glyphosate itself through fear of a further severe reaction being triggered.

The father's MEDs remained abnormal in February 2025, i.e., 2.5 years post‐incident (Table 1). Further monochromator testing in November 2025 showed normal MEDs, although he still had papular erythema following three consecutive days' BBUVA provocation (Figure 1f). This is in keeping with his clinical symptoms, which showed improvement over time, although with a milder rash still occurring during his last sunny weather exposure (August 2025). The child's MEDs were normal by July 2024, when 3 days provocation testing produced a borderline response; there was accompanying clinical improvement, with 2 mild episodes in 2025.

## 3 Discussion

Persistent/prolonged light reaction is a poorly understood phenomenon, which has been described after an initial episode of photosensitivity triggered by systemic and topical photosensitisers, such as quinine and ketoprofen, respectively [1, 2]. The pathomechanism is unknown, potentially involving immune dysregulation leading to a chronic photosensitive state [3].

Photosensitivity to glyphosate weedkiller is rare. We have identified one previous case in the literature of photosensitivity following contact with glyphosate formulation, where photopatch testing showed a phototoxic reaction to the coformulant BIT [4]. Instances of irritant contact dermatitis/chemical burns are also reported with glyphosate herbicides, although it's unclear whether adverse effects are attributable to co‐formulants [5]. Glyphosate itself is a phosphonic acid derivative, N‐(phosphonomethyl) glycine. To produce a phototoxic reaction, molecules generally require a level of unsaturation (double bonds/aromatic elements) and a metabolic/transportation profile producing sufficient quantity in skin, although many anomalies are observed to occur [6]. One human in vivo photopatch study, reported 1986, did not find evidence of photoirritation or photoallergy with glyphosate, but the sample size was relatively small to detect photoallergy [7]. More recently, in vitro studies evaluated the use of a new direct peptide reactivity assay, with UVA‐irradiation step, to assess the photosensitisation potential of six commercial glyphosate‐containing formulations [8]. Significant photosensitising properties were not detected, but it was recognised that the wide array of different glyphosate formulations in use may exhibit individual phototoxicity profiles [8].

Our patients potentially share a genetic profile resulting in immune responses predisposing to their prolonged photosensitivity reaction to the glyphosate product.

Management of the condition is supportive, comprising photoprotective measures and limitation of sunlight exposure, with topical anti‐inflammatory therapy for flares, and disease modifying agents can also be considered. For outdoor workers who may be at risk of higher and repeated exposure, minimisation of contact with glyphosate‐containing formulations through appropriate handling procedures and personal protective equipment is advised, and occupational modification may be required.

## 4 Conclusion

This case, incorporating detailed photobiological assessment, highlights a previously unrecognised phenomenon of prolonged light reactivity following exposure to glyphosate herbicide. Given its ubiquitous use and potential consequences for affected individuals, a rigorous evaluation of the photosensitising properties of glyphosate formulations, and their handling procedures, is warranted.

## Author Contributions

All authors take responsibility for the integrity of the data. All authors contributed to the manuscript conception, design, drafting and revision, and all authors read and approved the final manuscript.

## Funding

L.E.R. acknowledges the support of the NIHR Manchester Biomedical Research Centre (NIHR203308).

## Consent

Written patient consent for publication was obtained.

## Conflicts of Interest

The authors declare no conflicts of interest.

## Data Availability

The data underlying this article are available in this article.
